# Role of *ex vivo* Expanded Mesenchymal Stromal Cells in Determining Hematopoietic Stem Cell Transplantation Outcome

**DOI:** 10.3389/fcell.2021.663316

**Published:** 2021-05-04

**Authors:** Stefania Crippa, Ludovica Santi, Margherita Berti, Giada De Ponti, Maria Ester Bernardo

**Affiliations:** ^1^San Raffaele Telethon Institute for Gene Therapy, IRCCS San Raffaele Scientific Institute, Milan, Italy; ^2^Centro Ricerca M. Tettamanti, Department of Pediatrics, University of Milano-Bicocca, Monza, Italy; ^3^Pediatric Immunohematology and Bone Marrow Transplantation Unit, San Raffaele Scientific Institute, Milan, Italy; ^4^University Vita-Salute San Raffaele, Faculty of Medicine, Milan, Italy

**Keywords:** mesenchymal stromal cells, hematopoietic (stem) cell transplantation, hematopoietic stem and progenitor cell, bone marrow niche, immunoregulation

## Abstract

Overall, the human organism requires the production of ∼1 trillion new blood cells per day. Such goal is achieved via hematopoiesis occurring within the bone marrow (BM) under the tight regulation of hematopoietic stem and progenitor cell (HSPC) homeostasis made by the BM microenvironment. The BM niche is defined by the close interactions of HSPCs and non-hematopoietic cells of different origin, which control the maintenance of HSPCs and orchestrate hematopoiesis in response to the body’s requirements. The activity of the BM niche is regulated by specific signaling pathways in physiological conditions and in case of stress, including the one induced by the HSPC transplantation (HSCT) procedures. HSCT is the curative option for several hematological and non-hematological diseases, despite being associated with early and late complications, mainly due to a low level of HSPC engraftment, impaired hematopoietic recovery, immune-mediated graft rejection, and graft-versus-host disease (GvHD) in case of allogenic transplant. Mesenchymal stromal cells (MSCs) are key elements of the BM niche, regulating HSPC homeostasis by direct contact and secreting several paracrine factors. In this review, we will explore the several mechanisms through which MSCs impact on the supportive activity of the BM niche and regulate HSPC homeostasis. We will further discuss how the growing understanding of such mechanisms have impacted, under a clinical point of view, on the transplantation field. In more recent years, these results have instructed the design of clinical trials to ameliorate the outcome of HSCT, especially in the allogenic setting, and when low doses of HSPCs were available for transplantation.

## Introduction

The bone marrow (BM) is the organ responsible for hematopoiesis, the formation of blood cellular components. At the prenatal stage in humans, hematopoiesis is developed via interactions of hematopoietic stem and progenitor cells (HSPCs) with a stroma of mesenchymal lineage (Bianco et al., 2010). Postnatally, these interactions are carried out within the skeleton. Here, HSPCs are maintained, and hematopoiesis takes place, unless hematopoietic stress causes the process to transfer to extramedullary locations (Morrison and Scadden, 2014). Over the years, delineating a comprehensive picture of both the BM’s components and regulatory mechanisms have fascinated researchers, due to the potential of such discoveries to benefit the clinical practice, as for example in hematopoietic stem cell transplantation (HSCT). Furthermore, although the BM is constituted by multiple elements, summarized in Table 1, this review will revolve specifically around the role of the BM’s mesenchymal compartment, intended as mesenchymal stromal cells (MSCs) and MSC-derived osteoprogenitors/osteoblasts, given its fundamental role in the regulation of HSPC homeostasis.

**TABLE 1:** The BM niche components.

Type of cell	Role in the BM
Osteoblastic cells	The region lining the inner bone surface is enriched with osteoblastic cells, including osteoprogenitors, pre- and mature osteoblasts, and osteoclasts. The region is densely vascularized Le et al. (2018). Osteoprogenitors, the most immature precursors of the osteoblastic lineage, maintain HSCs and support their proliferation by forming supportive niches within the BM environment (Sacchetti et al., 2007). Osteoblastic cells are the primary contributors to the endosteal niche and were among the first cells to be identified to regulate hematopoiesis (Lord et al., 1975; Gong, 1978; Taichman and Emerson, 1994). The cells, activated by parathyroid hormone (PTH) or locally by PTH-related protein, have been observed to produce hematopoietic growth factor (Taichman and Emerson, 1994; Taichman et al., 1996). The number of osteoblasts, directly correlated with amount of HSC, is strongly influenced by bone morphogenic protein (BMP) signaling (Zhang et al., 2013), whose receptor inhibition leads to an increase of both osteoblasts and HSCs (Galán-Díez et al., 2018). Lowering in the amount of osteoblasts has previously shown a suppressing effect towards lymphoid and erythroid cells, whilst favoring myeloid cell expansion (Krevvata et al., 2014). Recent results have also suggested the degree of their involvement in hematopoiesis to depend on osteoblasts’ state of differentiation (Sacchetti et al., 2007; Wu et al., 2008; Mendez-Ferrer et al., 2010; Calvi et al., 2012; Kunisaki et al., 2013; He et al., 2017). Further evidence of the osteoblasts involvement in the regulation of HSCs self-renewal ability and correct homeostasis have been obtained in Calvi et al. (2003); Zhang et al. (2003).
Osteoclastic cells	Osteoclasts (OCLs), notoriously bone-resorbing cells and obligate partner of the osteolineage cells (Smith and Calvi, 2013), play a complex role in HSCs regulation, highly variable depending on the model. Studies have shown that administration of granulocyte-stimulating factor (G-CSF) promotes OCL activity in both humans and mice (Takamatsu et al., 1998; Watanabe et al., 2003). OCLs are fundamental for BM cavity formation, which, in turn, is necessary for HSPC mobilization (Katayama et al., 2006) and establishment of the HSC niche (Mansour et al., 2012). In detail, HSPC mobilization is triggered by OCL production of matrix metalloproteinase 9 and cathepsin K, which through cleavage of CXCL12, inactivates stromal cell-derived factor 1 (SDF-1) and causes HSPC to be released by the BM (McQuibban et al., 2001; Petit et al., 2002; Lévesque et al., 2003). However, results still appear conflicting on OCLs’ activities, with some studies showing OCL to actually inhibit mobilization in mice. This was theorized, when mice administered with bisphosphonate, which notoriously reduces the number of OCLs, were observed to have greater mobilization (Takamatsu et al., 1998; Winkler et al., 2010). Similarly, Miyamoto et al. (2011) observed greater mobilization when OCLs were depleted, and reduced following G-CSF serial administration and OCLs increase. Histological analyses further suggested that both OCL and the BM cavities may be negative regulators of hematopoiesis, making them unnecessary for HSPC maintenance or mobilization (Miyamoto et al., 2011).
Endothelial & Perivascular niche	Further components of the BM niche include perivascular HSCs, comprising endothelial and Leptin receptor positive (LepR^+^) stromal cells (Zhou et al., 2014). In Kiel et al. (2005) localized the perivascular niche in the sinusoidal endothelium, whilst endothelial cells (EC) line the lumen of blood vessels aimed at transporting oxygen and nutrients to cells. The endothelial niche can be further distinguished between an arteriolar and a sinusoidal niche, identified by distinctive markers (Lazzari and Butler, 2018). ECs contribute to HSCs regulation and self-renewal both directly or via the release of angiocrine factors, such as C-X-C motif chemokine 12 (CXCL12), VEGF-A, FGF2, ANG1, the Notch signaling pathway and TSP1 (Asada et al., 2017; Tikhonova et al., 2019). Moreover, a study by Chen et al. has highlighted the important regulatory role of a subpopulation of ECs, named Apln^+^ ECs, on HSCs correct functioning and BM regeneration following transplant (Chen et al., 2019). The BM endothelial components, comprise the arteriolar and the sinusoidal niches (Hooper et al., 2009; Poulos et al., 2015). In addition to these, another subset of cells, named ‘Type-H’ cells, and characterized by elevated CD31 and Endomucin, has been highlighted. Type-H cells are possibly widespread in both the sinusoidal and arteriolar niches (Ramalingam et al., 2017) and have been suggested to be significant contributors to regulation of the bone angiogenesis and BM’s microenvironment (Kusumbe et al., 2014; Ramasamy et al., 2014; Itkin et al., 2016). However, it has recently been suggested that quiescent HSCs are likely to reside in proximity of perivascular niche, closely associated to the sinusoid (Acar et al., 2015; Chow et al., 2011; Koechlein et al., 2016).
Neuronal & glial cells	In the last decade, the sympathetic nervous system (SNS) has been an adjunct to the many regulators for the BM’s HSCs, acting via direct contact, via the microenvironment or by releasing catecholamines (Kalinkovich et al., 2009). In particular SNS has been observed to regulate HSPCs’ mobilization from the BM to the bloodstream (Katayama et al., 2006; Méndez-Ferrer et al., 2008; Mendez-Ferrer et al., 2010) as well as their proliferation and differentiation. Recent findings have demonstrated the mechanisms of homing and mobilization of HSPCs to be regulated by coordinated activity of the SNS and parasympathetic cholinergic signals in García-García et al. (2019). SNS’s modulating influence of the BM have been evidenced in several processes, including bone remodeling, cellular anchorage and egress from the BM and HSPC inflammatory outcome (Hanoun et al., 2015; Maryanovich et al., 2018). Additionally, MSCs’ activity has been seen to undergo regulation by the sensorial nervous system and, in turn, secrete neurotrophic factors, which benefit the nervous system by exerting a neuroprotective effect and promoting regeneration, showing a reciprocal relation between the two systems (Teixeira et al., 2013). Moreover CXCL12 downregulation is achieved via noradrenaline binding to β 3-ADR located on stromal cells, which causes a decrease in intranuclear Sp1 transcription factor (Méndez-Ferrer et al., 2008; García-García et al., 2019). This regulatory mechanism has long been identified as essential to HSC/leukocyte preservation within the BM (Nagasawa et al., 1996). As part of the CNS involvement of the BM niche, glial cells have demonstrated a supportive role towards the BM nerve fibers in the regulation of HSCs’ proliferation, by maintaining HSCs’ quiescent state (Yamazaki et al., 2011). By releasing activator molecules, the tumor growth factor-β (TGF)/SMAD signaling pathways is activated and Smad2 and Smad3 phosphorylation is increased, guaranteeing HSCs’ quiescence (Yamazaki et al., 2011; Blank and Karlsson, 2015).
Adipocytes	Bone marrow adipocytes (BMAs) were first hypothesized in the 19th century and officially identified as BM components in the 1960s (Zakaria and Shafrir, 1967). BMAs are a heterogeneous group of cells both in terms of physical characteristics and varying among species and genders (Scheller et al., 2015; Lecka-Czernik et al., 2017). They are present on both the yellow and red BM and represent 50-70% of the total BM volume (Hardouin et al., 2016). Among the several different cell types making up the BM niche, BMAs play a significant role which, although previously only considered limited to a negative regulation (Naveiras et al., 2009), it has recently emerged as significant for HSCs appropriate regulation (Zhou et al., 2017). Imbalanced ratio of BMAs and a lower hematopoietic functional capacity has been associated with aging (Kirkland et al., 2002; Berkahn and Keating, 2004; Rosen et al., 2009; Van Zant and Liang, 2012). Although the basic function of BMAs is still to store excess energy, yellow and red BMAs have shown a difference in responsiveness (Craft et al., 2018). Studies have shown that BMAs from the yellow marrow do not respond to environmental stressors, whilst the red marrow mainly includes react to both endogenous and exogenous cues (Scheller et al., 2015). One of the mechanism through which BMAs affect hematopoietic stem cells’ function is by imposing a physical barrier when expanding in the BM. This causes a limitation in mobilization and a necessary remodeling of the marrow niche via lineage development, to adjust to the limited space available (Boroumand and Klip, 2020). Such limitations may further affect bone appropriate formation (Su et al., 2018) and alter lymphocytes’ maturation (Tokoyoda et al., 2004).
Macrophages	Macrophages are a heterogeneous group of mononuclear cells belonging to the innate immune cell group and are present across all tissue types, including the BM. They primarily act as a first line defense in immune response (Zajd et al., 2020) but exert several functions including tissue remodeling, clearing of dead cells and releasing of angiogenic factors (Davies et al., 2013). Macrophages form early in embryogenesis and are believed to migrate towards the sites of HSC formation to support their development (McGrath et al., 2015). Within the BM, macrophages interact with Nes+ cells, inducing CXCL12 transcription. Once macrophages deplete, CXCL12 expression is lost and HSC are released from the BM (Mariani et al., 2019). This process involves not only CXCL12, but also several other factors regulating HSC maintenance, such as ANGPT1 and vascular adhesion protein 1 (VCAM). Experimental depletion of monocytes and macrophages led to a decrease in BM CXCL12 and a selective inhibition of HSC maintenance genes, which caused HSC egression into the peripheral circulation (Chow et al., 2011). In the context of HSCT, macrophages are believed to perform a significant supportive role to guarantee reconstitution, although the mechanisms involved are still unclear (Qiao et al., 2018). Activation of BM macrophages following allogenic HSCT has been observed to impair correct recovery, increase mortality and lower overall survival, suggesting macrophages’ critical impact (Takagi et al., 2009). In mice, reduction in BM macrophages altered the proportion of BM-HSC and megakaryocytes, as well as white blood cells in the peripheral blood circulation. Such reduction could be associated with a number of damaging consequences, among which a delay in BM recovery from damage, an increase in apoptotic HSCs and a limited survival rate of sub-lethal dose irradiation mice confirming the essential role of BM macrophages in BM recovery (Ju et al., 2019).

*Table summarizing the non-hematopoietic components of the human BM niche.*

The first hypothesis of the existence of a hematopoietic niche dates back to 1978, when Roy Schofield theorized that a specific subgroup of cells within the BM was responsible for conserving HSPCs’ self-renewing capacity and preventing their uncontrolled activation (Schofield, 1978). In the same years, other groups were working to define the BM’s components exerting a nurturing effect on HSPCs. These works highlighted the primary role of BM osteoprogenitors in the regulation of HSPC homeostasis, by underlining HSPCs’ primitive tendency to concentrate toward the endosteal margins of long bones, in direct contact with osteoblasts supported by N-cadherin (Lord et al., 1975) (Gong, 1978#181). Later studies employing labeled immature cells confirmed the specific localization of long-term (LT)-HSPCs in endosteal sites (Nilsson et al., 1997) (Nilsson et al., 2001 #246) (Lo Celso et al., 2009) (Xie et al., 2009 #290). Further significant discoveries were made in the early 1990s, when osteoblasts were proven capable of producing hematopoietic cytokines and of exerting a supportive role toward primitive hematopoietic cells *in vitro* (Taichman and Emerson, 1994). In light of these findings, in the following years, several groups observed an impairment in the HSPC composition associated with disruption of the normal osteoblastic physiology. Accordingly, the stimulation of osteoblast proliferation increases the number of HSPCs in mice and *ex vivo* in co-culture setting (Raaijmakers et al., 2010). By providing evidence that osteoblastic cells played a critical role in HSPCs’ functioning (Calvi et al., 2003; Zhang et al., 2003; Sacchetti et al., 2007), studies in the early decades of the 2000s paved the way for further investigations delineating a highly multi-faceted picture. From that moment, different types of cells, either hematopoietic and have not been observed to be involved in BM’s appropriate functioning, were characterized more as complex systems than as simple groups of cells (Mendez-Ferrer et al., 2015). The concept arising from these studies highlights the perivascular localization of the BM niche, created partly by MSCs and endothelial cells, often associated with the sinusoidal vessels of trabecular bone (Morrison and Scadden, 2014). The role of osteoblasts in the control of HSPC hematopoiesis was revaluated in light of few HSPCs found in contact with osteoblasts, and further experiments demonstrated that osteoblasts do not impact significantly on HSPC proliferation and differentiation (Greenbaum et al., 2012). MSCs emerge as fundamental cells to physically support HSPCs and sustain HSPC self-renewal and differentiation to satisfy the body’s needs at physiological state and in the case of stress.

## Mesenchymal Stromal Cells

Mesenchymal stromal cells are key elements of the BM niche regulating the composition of the niche environment and the hematopoietic process. In support of this concept, several studies showed the co-localization of MSCs in the sites of hematopoiesis, starting from embryonic developmental stages E11 in the aorta–gonad–mesonephros and, following the development of the hematopoietic systems, in the liver, spleen, and BM (Mendes et al., 2005).

In order to better understand the complex interactions within the BM, mice of various strains have been extensively employed as models to study the human BM niche, revealing fundamental aspects of BM’s dynamic cellular activity. Such results, however, are based on a few marker genes, such as leptin receptor (*LepR*), nestin (*Nes*), C-X-C motif ligand 12 (*Cxcl12*), and Neural/Glia antigen 2 (*NG2*), which are not specific to the BM cells but are significantly expressed in other tissues as well (Chen et al., 2017). Nonetheless, given the important activities of these genes, their potential employment for therapeutic purposes have led scientists to investigate their characteristics. As an example, some *Nes*+ cells have been proven to originate from the neural crest, co-localize with HSPCs, and support HSPC functions in transgenic mice (Mendez-Ferrer et al., 2010; Isern and Mendez-Ferrer, 2011). More recently, *Nes*+ MSCs were characterized in the murine BM. These cells show a greater proliferative capability when compared with *Nes*- cells, together with an increased capacity to differentiate into mesodermal cells, release chemokines, specifically, colony-stimulating factor-1 (CSF-1) and tissue inhibitor of metalloproteinase (TIMP)-1 and -2 (Lu et al., 2019). Such characteristics are important to favor tissue regeneration following *Nes+* cell transplantation (Lu et al., 2019).

Similarly, Morrison and Scadden (2014) identified Leptin Receptor (*LepR*), a receptor for a fat cell-specific circulating hormone, as a marker to prospectively isolate mouse MSCs. *LepR+* MSCs show clonogenic capacity and localize around the sinusoidal vessels of the BM (Zhou et al., 2014). Importantly, *LepR*+ MSCs produce *Cxcl12* regulating HSPC homeostasis. In transgenic mice, the conditional reduction of *LepR*+ MSCs leads to a sharp decrease in Cxcl12, followed by a reduction of quiescent HSPCs and increased HSPC mobilization (Ding et al., 2012 #161; Ding and Morrison, 2013 #162; Oguro et al., 2013 #250). Finally, in the murine BM cavities, HSPCs show preferential proximity to CXCL12-abundant reticular (CAR) cells, surrounding the sinusoidal endothelial cells close to the endosteum (Sugiyama et al., 2006) as pericyte-like cells. As a matter of fact, *in vivo* analyses of HSPCs following transplantation in mice have provided significant evidence of their preferential localization in vessels displaying a higher rate of CXCL12, at first, and a subsequent migration toward periosteal locations (Sipkins et al., 2005; Lo Celso et al., 2009). CAR cells express adipogenic and osteogenic genes, and control HSPC homeostasis through the secretion of CXCL12 and SCF. Indeed, CAR cell-depleted mice showed an increased expression of myeloid-differentiation genes, highlighting the role of CAR cells in the control of HSPC quiescence (Omatsu et al., 2010). NG2 perivascular cells were also demonstrated to play a trophic effect on HSPCs. with arterioles. Depletion of NG2+ cells was associated with a sharp reduction in HSPC repopulating ability (Asada et al., 2017). Although differences between humans and mice should be taken into consideration, comparative biology has allowed the advancement in understanding the BM composition and mechanisms in this model, undoubtedly contributing to the current understanding of the interactions among human BM components and the supportive role that MSCs play in this context. Indeed, despite human MSCs showing different functional characteristics, the molecular mechanisms underlying the supportive activity of MSCs, first identified in the murine models of BM niche, were fundamental to dissect the complexity of the human hematopoietic niche. Recently, humanized models of the BM niche were employed as a tool to study the human stroma in physiological and pathological context, partially overcoming the functional differences between mouse and human BM niches (Reinisch et al., 2016; Abarrategi et al., 2017). Within the mature human BM niche, MSCs are localized around the blood vessels, similar to pericytes, in the endosteal and vascular sites (Mangialardi et al., 2016). The endosteal niche is localized in the internal bone shell surface, close to the endocortical and trabecular surfaces. The endosteum is a thin vascular membrane covering the inner surface of the bone mostly formed by osteoblasts and osteoclasts. Endosteal MSCs line the bone surface where they are physically associated with both osteoblasts and HSPCs (Mendez-Ferrer et al., 2010). Several studies have highlighted that long-term (LT)-HSPCs reside in the endosteal niche supported by endosteal MSCs. MSCs of the vascular niche consist of pericyte-like cells that support cycling HSPCs and regulate HSPC mobilization and homing. In particular, within the human BM niche, CD146^+^ MSCs are pericyte-like cells localized in the sinusoidal wall, characterized by high clonogenic and self-renewal capacities, in addition to expressing hematopoietic supportive factors, including SDF-1α and ANG1 (Sorrentino et al., 2008). CD146^+^ MSCs are capable of giving rise to hematopoiesis-associated stromal cells *in vivo*, with a defined developmental sequence in which bone formation precedes the appearance of a sinusoidal system, and ultimately of hematopoiesis (Sacchetti et al., 2007).

Contrarily, CD271 and CD271^+^/CD146^–/low^ MSCs are bone-lining MSCs associated with LT-HSPCs in low-oxygen areas. These specific subsets of MSCs are characterized by higher levels of extracellular matrix and chondrogenesis genes (Kuci et al., 2019). Also, CD271^+^ MSCs have shown high colony-forming unit—fibroblastic (CFU-F) capacity, the ability to transfer a BM microenvironment upon transplantation, and to differentiate *in vitro* into mesodermal mature cell lineages (Tormin et al., 2011; Crippa and Bernardo, 2018).

Although several studies have clarified the identity of MSC subset/s involved in the regulation of HSPC homeostasis *in vivo*, most of the available data describing their HSPC supportive activity have been obtained using *ex vivo* expanded MSCs. This is due to their low frequency in the BM of 0.01–0.001% of the total mononucleated cells in humans. Hence, MSCs are usually isolated and expanded *in vitro* to reach an appropriate quantity for biological and functional characterization, and an adequate number for any clinical application (Alvarez-Viejo et al., 2013). MSCs can be easily isolated from BM mononuclear cells by plastic adherence, and expanded as fibroblast-like cells for several passages (p8/9), with an intrinsic capacity to differentiate into mesodermal cell types when exposed to proper differentiation factors. Importantly, from a clinical point of view, it has been shown that MSCs can be also derived from the CD34- fraction of BM aspirates, highlighting the possibility to isolate autologous MSCs from the same sample used to isolate CD34^+^ HSPCs for transplantation (Ingo et al., 2016; Crippa and Bernardo, 2018).

Furthermore, MSCs have been isolated from several other tissues, including the umbilical cord (Erices et al., 2000), adipose tissue, liver (although with some differing characteristics) (Kholodenko et al., 2019), spleen, dental pulp, and lung tissue (Foronjy and Majka, 2012). To ease the identification of MSCs, in 2006, the International Society for Cellular Therapy has listed some defining criteria (Dominici et al., 2006):

(1) Adherence of MSCs to plastic when cultured.
(2) Expression of CD105, CD73, and CD90 surface markers and, at the same time, absence of expression of CD45, CD34, CD14/CD11b, CD79a, CD19, and HLA-DR surface antigens.
(3) Ability to differentiate *in vitro* in the three lineages: osteoblasts, adipocytes, and chondroblasts.

The clinical use of *ex vivo* expanded MSCs in the context of HSCT is based on MSC’s capacity to sense the environmental stress and activate a paracrine response to restore a proper balanced physiology. This includes the release of anti-inflammatory factors to preserve the BM niche function upon the pre-transplant treatment with conditioning regimen and the capability to modulate the innate and adaptive immunity, reducing the risk of graft-versus-host disease (GvHD) and graft rejection. In addition, the secretion of hematopoietic supportive factors by MSCs promote the survival of transplanted HSPCs and favor their engraftment, accelerating the hematological reconstitution after transplantation.

## Mesenchymal Stromal Cells’ Paracrine Immunoregulatory Activity

Over time, studies on cultured MSCs have attempted at clarifying MSCs’ regulatory role, and nowadays, their ability to produce and release a wide variety of cytokines, chemokines, and growth factors that may affect the immune system, has been extensively confirmed (Fibbe and Bernardo, 2014). MSC immunosuppressive ability was first demonstrated in support to skin graft, which was prolonged after MSC administration (Bartholomew et al., 2002). Thanks to this immunoregulatory capacity, MSCs may influence the activity of both the innate and adaptive immune cells, through cell-to-cell contact or by paracrine mechanisms. This renders MSCs a promising and attractive candidate for therapeutic application against different immune-mediated diseases and to control T-cell reactivity causing GvHD, in the case of allogenic transplants or inducing graft rejection.

The interaction between MSCs and the T-cell compartment has been demonstrated by several research groups. Francois et al. (2012b) reported that MSCs inhibit T-cell proliferation by secreting indoleamine 2,3-dioxygenase (IDO), especially when MSCs are pre-stimulated with IFNγ and TNF and when treated with prostaglandin E2 (PGE2). Moreover, it has been shown that the addition of monocytes to a PBMCs/MSCs co-culture increases the inhibitory effect of MSCs on T-cell proliferation in a dose-dependent manner (Cutler et al., 2010).

**FIGURE 1:**
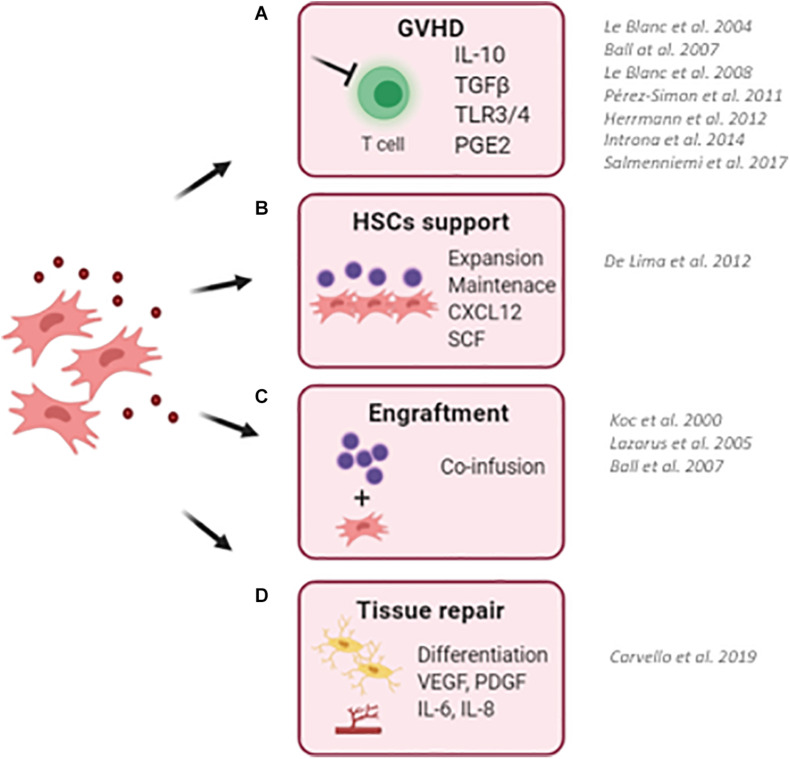
Schematic representation describing the clinical use of MSCs in the context of hematopoietic stem cell transplantation (HSCT). **(A)** MSCs has been successfully employed to reduce the risk and to treat graft-versus-host disease (GvHD) in transplanted pediatric and adult patients. Thanks to their ability to sense inflammatory stimuli, MSCs are capable to modulate T-cell proliferation and activation through the release of specific immunomodulatory cytokines (IL10, TGFb, and PGE2). **(B)** MSCs have been used as a feeder to expand and maintain UCB-CD34^+^ HSCs before transplantation due to their ability to secrete HSC supportive factors. **(C)** The co-infusion of MSCs has been demonstrated to promote HSC engraftment and accelerate hematological reconstitution in transplanted patients. Despite only a small percentage of infused MSCs reaches the BM niche, the production and release of supportive factors by co-infused MSCs ameliorates the outcome of HSCT. **(D)** The ability of MSC to repair and differentiate in bone cells makes them an attractive cell candidate to restore a proper BM niche, with MSCs and MSC-derived osteoblasts capable of supporting HSCs. (BM, bone marrow; UCB, umbilical cord blood). For each clinical application, works describing clinical trials for the use of MSCs are reported.

The mechanisms at the basis of T-cell immunosuppression rely also upon cytokines, such as interleukin-10 (IL-10), transforming growth factor-β (TGFβ) (Ryan et al., 2007), and also through toll-like receptors (TLRs), in particular, TLR3 and TLR4 (Liotta et al., 2008; Figure 1).

The immunoregulatory activity of MSCs is environment dependent, and MSCs acquire a specific function by sensing the inflammatory/anti-inflammatory signals in a process of activation named “licensing” (#97). The activation of immunosuppressive rather than immune-stimulating phenotype of MSCs depends on the balance between different stimuli. Based on these signals, MSCs can polarize into two acting phenotypes: MSC-1 with a proinflammatory profile and MSC-2 with an anti-inflammatory profile (Waterman et al., 2010). Among all factors, IFNγ is the key to activate MSC with immunosuppressive properties. IFNγ is the first cytokine to be produced after T-cell activation, and it would normally act as a boost signal for T cells. However, in the presence of MSCs, IFNγ suppresses T-cell proliferation, by binding IFNγ receptors expressed on the cell surface of MSCs and activating an immunoregulatory phenotype of MSCs. It has also been shown that MSCs treated with IFNγ *in vitro*, behave as an efficient antigen-presenting cell (APC), inducing specific immune responses. It was suggested that MSCs act in different ways depending on the concentration of IFNγ: they may act as APCs at low IFNγ concentration, while exerting an inhibitory effect at higher concentration of IFNγ (Chan et al., 2006). The release of other inflammatory cytokines, such as TNF-α and IL-1a, are important for MSC activation, but the effect of these stimuli is only effective in combination with IFNγ.

An important role for the activation of immunosuppressive MSCs is played by TLRs. MSCs express different types of TLRs, which trigger specific molecular signaling and biological properties. For example, TLR2 induces the secretion of IL-6 and inhibits differentiation of MSCs into adipocytes and osteoblasts (Flynn et al., 2019); TLR3 induces MSC migration and secretion of immunosuppressive cytokines and chemokines like IL-10 and TNFα (Rashedi et al., 2017). On the contrary, TLR4 priming induces MSCs to release proinflammatory cytokines. In conclusion, the licensing process depends on three factors: presence of inflammatory cytokines, their concentration, and timing of exposure (Romieu-Mourez et al., 2010; Sangiorgi and Panepucci, 2016).

Furthermore, MSCs have shown the ability to variably respond to IFN-γ depending on the circumstances. For example, IFN-γ may enhance the immunosuppressive activity of MSCs on T lymphocytes (Krampera et al., 2003), but it may also elicit a completely different response, by inducing MSCs to behave as a non-conventional antigen-presenting cell (APC) (Stagg et al., 2006), demonstrating a high immunological plasticity. While effects on T cells is clearer, regulation of B-cell function remains controversial. Several studies show inhibition of B cells, through cell cycle arrest elicited by MSCs, mainly due to the production and release of soluble factors (Augello et al., 2005; Corcione et al., 2006). This aspect supports the prospective employment of MSCs in immune-mediated disease. Conversely, Healy et al. have shown a completely opposite effect, as MSCs determined activation and proliferation of B lymphocytes (Healy et al., 2015), highlighting the necessity to continue the research to improve our understanding of the underlying regulatory mechanisms.

Importantly, although the mechanisms still need to be clarified, these cells have shown fundamental immunoregulatory capacity by significantly affecting maturation, polarization, and function of lymphocytes, macrophages (Zhou et al., 2019), dendritic cells’ (DCs), and natural killer (NK) cells (Sotiropoulou et al., 2006).

The immunomodulatory ability of MSCs is at the basis of their clinical use in promoting the resolution of damage-induced inflammation. In this regard, MSCs not only modulate the immune system to mitigate the detrimental effects of excessive inflammation, but also release specific factors within the inflammation site, which favor resident tissue repairing mechanisms. Among them, VEGF, Angiogenin (ANG), and IL-8 increased vascular regeneration (Kim et al., 2019), NGF, IL-10, and IL1-RA prevented apoptosis and enhanced cell proliferation (Francois et al., 2012b). Stimulation of MSCs with TNF, for example, has been observed to enhance the release of interleukin 6 (IL6), HGF (Broekman et al., 2016), VEGF (Ge et al., 2018), and insulin-like growth factor-1 (IGF-I), which, on the other hand, is decreased when p38 MAPK signaling pathway is halted (Miloso et al., 2008). These mechanisms have shown potential to be employed in treatment. For example, combinatory effect of p38 MAPK inhibitors with MSC infusion has recently been tested in mouse models of myocardial infarction, significantly ameliorating inflammation, apoptosis, and cell morphology (Zhang et al., 2017), highlighting the beneficial effect of this combinatory strategy to limit inflammation-derived damage. Similarly, MSCs have been employed in several clinical trials to counteract neurodegenerative diseases (Kabat et al., 2020), showing neuroprotective effects. Indeed, in Parkinson’s disease, hBM-MSCs promoted a-synuclein clearance stimulating IL-4 secretion from microglial cells, (Park et al., 2016) and in Alzheimer’s disease, hUCB-MSCs were able to reduce microglial activation and apoptosis (Lee et al., 2010).

## Mesenchymal Stromal Cells’ Hematopoietic Support

**FIGURE 2:**
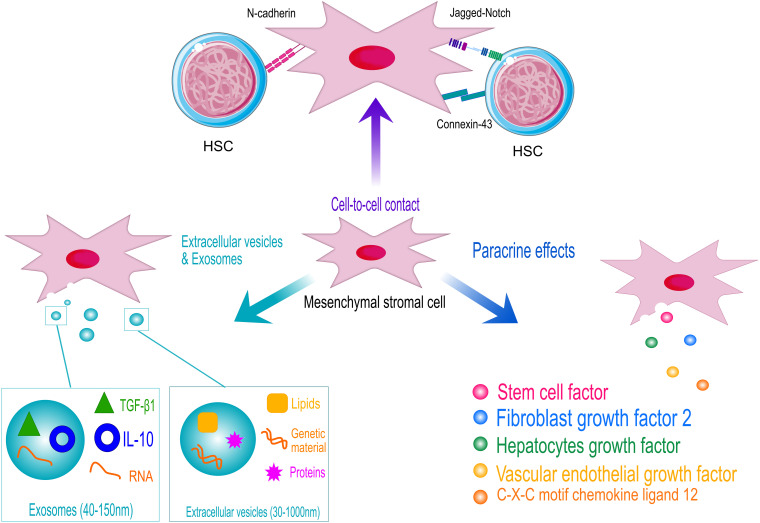
Schematic representation of the hematopoietic supportive activities of human mesenchymal stromal cells (MSCs). MSCs support hematopoietic stem and progenitor cell (HSPC) homeostasis by cell-contact, through the interaction of specific ligands expressed on MSCs surface with HSPC receptors (N-cadherin and Notch). Adherent junction, such as connexin43, also play a role in the control of HSPC metabolism to protect cells from excessive activation. However, MSCs exert their hematopoietic function mainly through the secretion of supportive factors and the release of extracellular vesicles and exosomes. HSC models are licensed by https://creativecommons.org/licenses/by/3.0/legalcode.

The key role of MSCs in the regulation of the hematopoietic compartment is supported by the co-localization of MSCs with sites of hematopoiesis since the embryonic developmental stages (Mendes et al., 2005). MSCs exert their function through the direct interaction with HSPCs and by secreting several paracrine factors, as schematized in Figure 2. Perivascular MSCs express several Notch ligands, including Jagged-1, Jagged-2, DLL-1, and DLL-4, which are responsible for the activation of Notch signaling in HSPCs, a key pathway controlling HSPC growth and differentiation during development (Bigas et al., 2010, #345; Lampreia et al., 2017). The perturbation of Notch ligand expression in MSCs induces premature differentiation of HSPCs when *ex vivo* co-cultured (Corselli et al., 2013).

Within the BM niche, the levels of ROS determine the balance between quiescent and differentiating HSPCs. Thus, the fine tuning of ROS level is fundamental to preserve HSPC homeostasis (Ludin et al., 2014). In such a context, MSCs function as scavenger cells to import ROS from HSPCs avoiding excessive HSPC activation (Taniguchi Ishikawa et al., 2012). Similarly, it has been shown that the mitochondrial transfer from transplanted HSPCs to BM stroma via connexin-43 accelerate the BM niche regeneration after myeloablative stress, improving HSPC engraftment (Golan et al., 2020). The N-cadherin-mediated binding of human HSPCs to BM stromal cells was also described to preserve HSPC quiescence. *In vitro* studies demonstrated that N-cadherin silencing reduces the percentage of long-term repopulating HSPCs in co-culture with MSCs (Wein et al., 2010). In addition, the contact-dependent effects of MSCs on different BM niche cell types allow to amplify the supportive activity of the stromal niche. For instance, the direct cell contact between MSCs and endothelial cells induce MSCs to activate a pericyte-like program to sustain the formation of new vessels (Loibl et al., 2014) and the integrin-mediated cell-contact of MSCs with myeloma cells enhance the production of osteoclast-stimulating factors (Michigami et al., 2000).

However, the MSC release of supportive factors is the principal molecular mechanism regulating HSPCs\homeostasis *in vivo*, within the BM niche, and *ex vivo*, in MSC-based co-culture systems and when MSCs are co-infused to sustain HSPCs in HSTC pre-clinical and clinical models (Crippa et al., 2019). The role of MSC secretome (Liu et al., 2018) and MSC-derived extracellular vesicles (Batsali et al., 2020) also demonstrates the predominance of MSC paracrine activity in sustaining HSPC function.

Among the many components of MSCs’ secretome, CXCL12, also known as stromal cell-derived factor-1α (SDF-1α), and stem cell factor (SCF), or Kit ligand (Kitl), play a pivotal role in HSPCs’ maintenance. CXCL12 has shown both a significant immunomodulatory effect (Giri et al., 2020) and an essential regulatory activity on both HSPCs and lymphoid progenitor cells (Semerad et al., 2005; Mendez-Ferrer et al., 2010). Suppression of *Cxcl12* from multiple subgroups of stromal cells was observed to cause a range of effects in terms of mobilization and appropriate functioning of cells. Interestingly, while no effect was observed when Cxcl12 was deleted in mineralizing osteoblasts, B-cell progenitors were lost and HSPC mobilized, when the same factor was deleted from osterix-expressing stromal cells (Greenbaum et al., 2013). Furthermore, when Cxcl12 was deleted in murine *Nes-* mesenchymal progenitors, a striking loss of HSPCs and quiescence as well as long-term repopulating activity and common lymphoid progenitors were observed, highlighting this subpopulation of cells to retain a supportive role of B-cell progenitors and its significance in HPSC homing (Greenbaum et al., 2013). CXCL12 selectively activated STAT-5 signaling pathway in different hematopoietic cells, inducing cell proliferation (Vila-Coro et al., 1999; Mowafi et al., 2008). The activation of STAT5 in HSPCs is accompanied with the expression of specific microRNAs to guarantee a negative control for excessive and uncontrolled HSPC proliferation (Haetscher et al., 2015), suggesting a possible role of CXCL12/STAT5 axis in HSPCs’ survival and proliferation (Janowska-Wieczorek et al., 2001; Aqmasheh et al., 2017).

Mesenchymal stromal cells’ secretion of SCF has been instead associated with appropriate homing of HSPCs (Horwitz et al., 2011). Mainly secreted by BM adipocytes and *Lepr+* stromal cells, studies on mice have proven that SCF is essential for HSPC regeneration as well as hematopoiesis and cell homeostasis, particularly in conditions of obesity and aging (Zhang et al., 2019; Zhou et al., 2017). Inhibition of the appropriate interactions between SCF receptor and ligand has shown to enhance HSPC clearance, confirming the chemokine role in HSPCs’ self-renewal (Czechowicz et al., 2007). Evaluation of mutations to the SCF receptor-encoding genes in mice models have been associated with severe conditions of macrocytic anemia, in the case of loss-of-function mutations (Nocka et al., 1989), or erythrocytosis when in the presence of a gain of function (Bosbach et al., 2012).

Among the many components of MSCs’ secretome, several were observed to exert important roles in inflammatory reactions, cell homing, and regulation of apoptotic events, such as vascular endothelial growth factor (VEGF), fibroblast growth factor-2 (FGF-2), angiopoietin (ANGPT), and hepatocyte growth factor (HGF), all associated with different signaling pathways (Aqmasheh et al., 2017).

Vascular endothelial growth factor is another factor secreted by MSCs and involved in several immunological responses, enhancing neuro- and angiogenesis in cases of cerebral damage (Matsuzaki et al., 2001; Svensson et al., 2002; Ford et al., 2011; Suzuki et al., 2011). VEGF is also a crucial regulator of cell differentiation by determining cell fate as well as survival, migration, and proliferation (Oswald et al., 2004; Li et al., 2011; Zaniboni et al., 2015). In terms of hematopoietic support, VEGF levels in HSPCs tend to raise in response to stimulation by cytokines (Bautz et al., 2000). Several subsets of HSPCs present VEGF receptor type 2 (VEGFR-2), whose expression has been linked to pluripotent stem cell activity (Shalaby et al., 1995; Kabrun et al., 1997). At the end of the last century, early development studies observed lethality due to alteration in both angiogenesis and hematopoiesis, as a consequence of knockout of either VEGF- and VEGFR-2-encoding genes (Shalaby et al., 1995; Carmeliet et al., 1996; Ferrara et al., 1996). In particular, Shalaby et al. (1995) observed Vegfr2^–/–^ mice dying around E8.5. Similarly, knock-in mice with tyrosine residue Y1173 of *Vegfr-2* (Y1175 in humans) were observed to die around E8.5–9.5, due to scarcity of endothelial cells and HSPCs (Sakurai et al., 2005). This is due to VEGF ability in recruiting HSPCs and endothelial progenitor cells, which leads to the development of microvasculature within the BM, essential for appropriate hematopoiesis (Gerber et al., 2002; Koch and Claesson-Welsh, 2012). Also, VEGF-A levels have been shown to increase in plasma as a result of ANGPT-1 stimulation of hematopoiesis and mobilization of BM-repopulating stem and progenitor cells (Hattori et al., 2001; Kopp et al., 2006). Although some of VEGF abilities to support hematopoiesis have been individuated, the underlying mechanisms that govern the process still need to be unveiled, and further studies are required.

Another example of the regulatory role of MSC’s secretome includes the maintenance of HSPCs’ quiescence regulated by interaction between tyrosine kinase receptor (Tie2) and ANGPT-1 ligand, through adhesion of MSCs to HSPCs (Blank et al., 2008). Similarly, HSPCs are maintained by MSCs’ production of the Notch ligands, which are normally involved in regulation of the proliferation, functioning, and differentiation of T and B lymphocytes (Kang and Robling, 2014). Production of Notch ligands improves survival and proliferation of HSPCs, while preventing their differentiation, as demonstrated by Notch inhibition leading to greater egress and mobilization of HSPCs (Wang et al., 2015).

Another significant player in HSPC maintenance is fibroblast growth factor 2 (FGF2) that has been demonstrated to support the expansion of stromal cells in mice, consequently raising SCF and supporting HSPC expansion, which highlighted the capacity of FGF2 to regulate gene expression and, in turn, support HSPC proliferation. In mice, FGF2 has been proven to promote appropriate recovery, following myeloablative treatments, by supporting HSPC expansion (Itkin et al., 2012; Zhao et al., 2012). Nonetheless, FGF2 ability to expand different types of progenitor cells may be crucial to boost hematopoiesis after HSCT.

Similarly, hepatocyte growth factor (HGF) is known to stimulate local angiogenesis by stimulating tyrosine phosphorylation via the c-MET receptor (Nita et al., 2017). HGF is produced within the BM microenvironment and has been observed to raise in patients treated with granulocyte colony-stimulating factor (G-CSF). Increased expression of the c-MET, associated to HGF, is observed in more mobile HSPC, concordant with the previous findings stating HGF/c-MET pathways to be associated with MSC mobilization. Hence, HGF and G-CSF may both be involved in establishing a proteolytic microenvironment in the BM, easing the egress of HSPC in peripheral circulation (Jalili et al., 2010).

Finally, another method through which MSCs interact, modify, and respond to the surrounding environment is by releasing extracellular vesicles (EVs). EVs are membrane-bound particles, ranging between 30 and 1,000 nm of dimension, that contain and transport biomolecules such as lipids, proteins, and genetic material between cells. When secreted by MSCs (MSC-EV), these particles mediate the cells’ paracrine activity and have been observed to exert an impactful anti-inflammatory effect in a number of conditions, including post-transplant complications, in addition to control normal and pathological hematopoiesis (Batsali et al., 2020; Lia et al., 2020). Under a clinical point of view, EVs appear promising due to their highly safe profile, low immunogenicity, and ability to cross biological barriers, given their attractive characteristics (Park et al., 2019). In hematopoiesis, MSC-EVs are believed to contribute to activate HSCs in response to different stimuli, including blood hemorrhage, fluctuations in oxygen concentrations, radiation, or chemotherapy (Butler et al., 2018). *In vitro*, MSC-EVs have been shown to promote the proliferation of umbilical cord-derived HSCs by triggering the Wnt/β-catenin pathway as well as to lower radiation damage to murine HSCs by promoting their proliferation (Cominal et al., 2019). The translation of such results into clinical practice, however, will still require extensive additional investigation to address significant limitations surrounding EVs’ production, quantification, pharmacokinetics, and characterization (Gowen et al., 2020).

Among the different kinds of EVs, exosomes are membrane-bound nanoparticles (40–150 nm) released by multivesicular bodies. MSC-derived exosome contains several bioactive molecules, particularly miRNAs of different kinds, plays a critical role in the control of both physiological and pathological states, including inflammatory response (Burrello et al., 2016) but also tumorigenesis and progression (Ono et al., 2014).

In light of such prominent functions exerted by MSCs in the context of the hematopoietic process, the different pathways and mechanisms are now under critical evaluation in order to better understand the homeostatic supportive process and potentially exploit certain mechanisms for therapeutic purposes. One of the main settings that could benefit the most is that of hematopoietic stem cell transplantation (HSCT).

## Clinical Applications Exploiting the Immunoregulatory Activity of Mesenchymal Stromal Cells

The paracrine activity of MSCs has rendered these cells an attractive therapeutic tool for several clinical applications, considering the versatile function of MSCs in different inflammatory and regenerative contexts. It has been a long time from the first MSC injection in human subjects (Lazarus et al., 2005), demonstrating that the use of MSCs is feasible and safe. Several clinical trials employing MSCs have been opened to counteract chronic degeneration, with particular interest for neurological disorders, to repair damaged tissues, and to mitigate the inflammatory response in autoimmune diseases and GvHD, in the context of HSCT.

In addition to the *in vitro* evidences demonstrating their immunomodulatory properties, MSCs have also been employed *in vivo* to suppress an excessive activation of the immune response in different clinical contexts, such as HSCT, tumor immunity, and autoimmunity, occasionally producing conflicting results. Substantial beneficial effects of MSC administration have been obtained in an autoimmune encephalomyelitis model, an autoimmune inflammatory disease that affects the CNS mediated mainly by T cells and macrophages (Zappia et al., 2005). Another inflammatory condition that might take advantage of MSCs infusion is Crohn’s disease (CD). MSCs immunomodulatory properties are, in fact, believed to reduce the persistent inflammation of the gastrointestinal tract by secreting anti-inflammatory molecules in the target tissues and modulating specific immune cells, for example, upregulating Treg cells, a cell population well known to be reduced in CD (Dalal et al., 2012; Carvello et al., 2019). Conversely, in another autoimmune condition, collagen-induced arthritis, MSCs injection, which is supposed to inhibit T-cell proliferation, did not lead to any significant benefit on arthritic symptoms. However, *in vitro* TNFα addition was sufficient to reverse MSC immunosuppressive effects, suggesting that environmental parameters and the microenvironment, in general, may influence and affect the immunomodulatory properties of MSCs (Djouad et al., 2005). Still, MSC treatment in humans has shown promising outcomes in several conditions, including GvHD (Le Blanc et al., 2004) and engraftment promotion (Lazarus et al., 1995; Koc et al., 2000), thanks to their potent immunosuppressive role but also in some rare diseases in which MSCs may be responsible of ameliorating the disease pathology in specific tissues (Crippa et al., 2019). Therefore, a better understanding of MSCs’ immunomodulatory role might be crucial to develop novel and effective therapeutic strategies for a broad range of diseases.

## The Clinical Role of Mesenchymal Stromal Cell in Hematopoietic Stem Cell Transplantation: Treatment of Graft-Versus-Host Disease

Considering their immunoregulatory and hematopoietic supportive function, MSC-based cell therapy has been employed to ameliorate the outcome of HSCT.

One of the most severe complications following allogeneic HSCT, is the development of acute GvHD (aGvHD), contributing to the high incidence of transplant-related morbidity [Szyska and Na, 2016 #123].

Both acute and chronic GvHD, occurring in the post-transplant period, may be treated with corticosteroids, considered the first choice therapy [Garnett et al., 2013 #89]. However, as patients may become resistant and may not benefit from this treatment, steroid-resistant GvHD (SR-GVHD) still lacks a common standardized treatment (Wolf et al., 2012). Hence, significant efforts toward new therapies, addressing this unmet clinical need, may overcome these immune-mediated disorders. Second-line treatments for SR-aGvHD have been developed by The American Society of Blood and Marrow Transplantation and mainly include compounds with immunomodulatory activity and monoclonal antibodies (Martin et al., 2012). Among recent treatment strategies, the better understanding of the immunomodulatory hallmarks displayed by MSCs sustains the rationale of MSC-based therapy for immune-mediated diseases, including aGvHD. As MSCs might be able to leave the circulation and reach damaged tissues, they may improve local lesions, caused by aGvHD. Moreover, as the paracrine effect of MSCs is one of the central mechanisms responsible for their clinical benefits, the use of extracellular vesicles (EVs), released by MSCs, has been investigated as potential therapeutic medical product, compared with cell therapy (Wang et al., 2012; Rani et al., 2015). In the clinical setting, MSC-EV infusion has been demonstrated to significantly ameliorate GVHD symptoms in a steroid-resistant patient. The anti-inflammatory molecules, carried by EVs, IL-10, TGFβ, and HLA-G, might be responsible for the beneficial effects of MSC-EVs as anti-inflammatory mediators (Kordelas et al., 2014).

In mice, the infusion of MSCs, derived from different sources, has shown a great immunomodulatory potential to control lethal GvHD (Yanez et al., 2006). However, contradictory results were obtained in another study by Sudres et al. (2006) in which MSC administration did not ameliorate GvHD. Further studies, aimed at improving the clinical use of MSCs in the context of GvHD suggested that multiple MSC injection (Tisato et al., 2007) could be necessary to prevent the disease onset in a murine model of aGvHD. This highlights the need to better define the therapeutic window for MSC administration and, eventually, to determine whether multiple injection of MSCs could improve their therapeutic efficacy.

In patients, MSCs were successfully employed for the first time by Le Blanc et al. (2004) to treat GvHD. The intravenous infusion of maternal BM-derived MSCs was able to control refractory aGvHD manifestations. The same authors reported in a phase II clinical trial that the infusion of *ex vivo* expanded BM-MSCs significantly improve the disease progression in 55 transplanted patients affected by steroid-resistant aGvHD. Thirty of the treated patients showed a complete remission, and in nine patients, symptoms improved, demonstrating that MSC administration represents a safe and effective treatment option for aGvHD. Any toxicity related to the injection of MSCs was reported (Le Blanc et al., 2008). Several additional clinical studies demonstrated the beneficial effect of MSC infusion against steroid-resistant aGvHD (von Bonin et al., 2009) in adults and in pediatric patients, with high responses rate in children (Ball et al., 2007; Salmenniemi et al., 2017).

Although the immunoregulatory effects of MSCs have been well established for the treatment of aGvHD, the efficacy of MSCs in the context of chronic GHvD (cGvHD), occurring 100 days after allogeneic HSCT and responsible for the late mortality of transplanted patients, is poorly characterized. Intra-BM injection of BM-derived MSCs were performed in four sclerodermatous cGvHD patients showing a gradual symptom improvement, although a complete response was not achieved (Zhou et al., 2019). However, in a prospective study, Peng et al. reported a marked amelioration of cGVHD manifestations in 23 patients after BM-MSC infusion, mainly through an increased number of IL10-producing CD5^+^ B-cells (Perez-Simon et al., 2011; Herrmann et al., 2012; Introna et al., 2014; Peng et al., 2015).

Despite the infusion of MSCs that has been demonstrated to be effective in the treatment of GvHD in Phase I/II clinical trials of allogenic transplants, the use of MSCs as a medical product to ameliorate aGvHD following HSCT remains controversial due to conflicting results achieved in different clinical trials. A phase III clinical trial carried out in the United States failed to match the expected primary endpoint to demonstrate the immune suppressive function of MSCs to treat GvHD (NCT00366145, 2020). Such conflicting outcomes induced the scientific community to consider the differences between the industrial and academic MSC-based products, which were evaluated in detail by Galipeau (2013), suggesting some levels of variance in MSC epigenetics, immunogenicity, and methods of preservation. From the first industrial phase III clinical trial (NCT00366145) to treat aGvHD, two commercial MSC products reached clinical approval and are on the market (Prochymal and Temcells). Hence, while some countries such as New Zealand and Canada have approved an MSC-based therapeutic drug (Prochymal^®^), as well as Japan (TEMCELL) for steroid-resistant patients, others, including the United States and China, have not (Galipeau, 2013; Zhao et al., 2019). Recently, other MSCs sources have been under investigation. Although MSCs isolated from BM are the most preferred source, mainly because they are most well-studied, a phase I/II clinical trial (NCT02172937) evaluated the efficacy of placenta-derived decidua stromal cells (DSCs) to treat aGVHD.

## The Clinical Role of Mesenchymal Stromal Cell in Hematopoietic Stem Cell Transplantation: Hematopoietic Stem and Progenitor Cell Engraftment

Besides GvHD, graft failure is one of the main complications associated with HSCT, caused by the activation of an immunologic response against the transplanted cells, in the case of mismatched donors, or by a reduced level of HSPC engraftment, especially when a low number of HSPCs are available for transplantation (Locatelli et al., 2014). This latter is a common event in the context of umbilical cord blood (UCB) HSCT, due the low frequency of CD34^+^ in UCB. Considering their immune-regulatory function and their HSPC supportive role within the human BM niche, MSCs have been proposed as co-adjuvant cells to control the recipient immune system and to sustain the engraftment of transplanted HSPCs. Moreover, with the aim to increase the number of transplanted HSPCs, MSCs have been employed *in vitro* to expand UCB CD34^+^ before administration, improving the hematological recovery of transplanted patients. In this specific context, the use of an MSC feeder has been shown to enhance HSPC expansion, a fundamental requirement for the engraftment of LT repopulating HSPCs. MSCs exert their function as supportive feeder through the secretion of several supportive and anti-inflammatory factors (Wagner et al., 2007; Crane et al., 2017), capable of stimulating HSPC proliferation while avoiding excessive HSPC culture-induced activation (Robinson et al., 2006; Wagner et al., 2007). The number of UCB-HSPCs available for transplantation was significantly higher when HSPCs were cultured in the presence of MSCs compared with the classical liquid culture expansion method (Robinson et al., 2006). Importantly, a phase I clinical trial demonstrated that the infusion of UCB-HSPCs, co-cultured *in vitro* with MSCs, is a safe and effective procedure to improve HSPC engraftment, being associated with faster neutrophil and platelet engraftment in transplanted patients (de Lima et al., 2012). The rationale behind the employment of MSCs in support of HSPC expansion and maintenance is the attempt to reproduce *in vitro* the supportive interactions with the BM stromal compartment, which regulate HSPC homeostasis. The capacity to facilitate HSPC expansion *in vitro* has been also described for MSCs isolated from different sources (Kadekar et al., 2015). Furthermore, not only have MSCs been exploited for HSPCs support, but also their differentiated osteoblast counterpart has been demonstrated to be effective for HSPCs *in vitro* maintenance and expansion. As an example, BM MSC-derived osteoblasts have been shown to promote HSPC expansion and cell growth compared with feeder-free cultures (Alsheikh et al., 2017; Michalicka et al., 2017). Moreover, transplantation experiments revealed that osteoblasts co-cultured with HSPCs was associated with enhanced long-term HSPC engraftment *in vivo*. Besides, Michalicka et al. found that the mechanism at the basis of growth-promoting cells is elicited by the β-catenin signaling pathway, as β-catenin inhibition profoundly reduced HSPCs progenitors’ growth (Michalicka et al., 2017).

In addition to the use of MSCs as a feeder to expand HSPCs before transplantation, MSCs have been infused in pre-clinical models and clinic trials of HSCT to favor the engraftment of HSPCs. MSCs sustain HSPCs mainly through the secretion of hematopoietic supportive factors that are released by co-infused MSCs to favor HSPC engraftment. Several pre-clinical studies have demonstrated that the co-infusion of third party fetal- and adult-derived MSCs enhance HSPCs engraftment, increasing the rate of HSCT success (Almeida-Porada et al., 2000; Noort et al., 2002). It has been shown that an increased presence of human circulating CD45+ cells at early time points after transplantation in mice co-infused with MSCs and an enhanced hematopoiesis in the BM at later time points in the presence of MSCs (Almeida-Porada et al., 2000). Interestingly, the supportive effect of MSCs is more robust when a limited dose of CD34^+^ is transplanted. In this case, the co-infusion of MSCs resulted in a threefold increase in HSPC engraftment in the BM of transplanted mice (Noort et al., 2002). The clinical benefit of MSC co-transplantation has been also shown in the autologous setting both in mice (Fernandez-Garcia et al., 2015) and in non-human primates (Masuda et al., 2009). Moreover, it is important to consider that the BM stromal niche might be damaged by the conditioning regimen and could be impaired in patients affected by specific diseases, including rare genetic diseases (Amarachintha et al., 2015; Starc et al., 2017; Crippa et al., 2019a) and hematological malignancies (Tian et al., 2011). The work of Abbhuel et al. demonstrated that the BM stroma is severely and long-term damaged by the conditioning regimen and supported the need to restore stromal function to improve the HSCT outcome (Abbuehl et al., 2017). In this light, the co-infusion of third-party MSCs may represent an attractive option to reach this objective. In regard to intrinsic stromal impairment, a reduced hematopoietic supportive capacity has been observed in MSCs derived from β-thalassemia patients, due to accumulation of high level of reactive oxygen species (ROS) (Crippa et al., 2019a). Similarly, the immune-regulatory capacity of MSCs isolated from patients affected by primary immunodeficiencies was impaired compared with healthy controls (Starc et al., 2017). MSCs isolated from AML and ALL patients showed several degrees of abnormalities (Tian et al., 2011). Moreover, it must be taken into account that conditioning regimens prior to transplantations perturbed the homeostasis of the BM niche, triggering a variety of cellular responses that may influence the outcome of transplantation (Ganuza and McKinney-Freeman, 2017). In this regard, IL-1 has been demonstrated to play a fundamental role after BM injury. It acts as an inflammatory marker by promoting myeloid differentiation and recovery of HSPCs after BM damage such as myeloablation prior to transplantation (Pietras et al., 2016). IL-1 could be seen as a mediator that provides communication between the inflammation status and HSPCs and the BM niche.

These data highlight the importance of a proper BM microenvironment to favor HSPC engraftment that should be restored in case of damage for a successful HSCT.

The paracrine activity of co-infused MSCs facilitates HSPC engraftment by providing a plethora of supportive factors, which compensates possible alterations in the resident BM stromal cells. In light of this, the co-infusion of MSCs genetically engineered to express growth factors significantly improved HSPC engraftment. In particular, an enhanced human HSPC engraftment was observed in a xenograft model of transplantation when MSCs overexpressing PDGFB were co-infused with HSPCs (Yin et al., 2020). PDGFB is known to modulate endothelial cell proliferation and promotes vessel regeneration, which is fundamental to restore a proper BM vascular network (Battegay et al., 1994). Indeed, Chen Q and colleagues demonstrated that radiotherapy and chemotherapy damage the BM vascular network. This affects the capacity of transplanted HSPCs to reach their hematopoietic niche and correctly engraft. They also showed that VEGF-A promotes the normalization of bone vasculature acting on Apln+ECs, which are critical for the maintenance of steady-state hematopoiesis (Chen et al., 2019). In such a context, VEGF was associated with reduced severity of aGvHD and mortality in patients undergoing allogenic HSCT (Min et al., 2006). In front of these evidences, we may consider that, when co-transplanted, MSCs indirectly sustain HSPC engraftment promoting the regeneration of a proper vasculature network through the secretion of VEGF, in addition to directly promoting HSPC survival. The supportive role of MSCs has been also demonstrated in clinical trials of MSC co-infusion. The majority of the data have been obtained co-infusing the third-party MSCs. MSCs do not express class II MHC, avoiding immune rejection (Ryan et al., 2005). The results of these studies highlighted an enhanced HSPC engraftment and hematological reconstitution when MSCs where co-infused with HSPCs. In the first clinical trial using MSCs, 28 breast cancer patients were co-infused with autologous HSPCs and 1–2 × 10^6^/kg *ex vivo* expanded MSCs showing no toxicities and a rapid hematopoietic recovery (Koc et al., 2000). Similarly, in an open-label, multicenter trial, 1–5 × 10^6^ MSCs/kg were infused 4 h before the administration of BM- or peripheral blood-derived HSPCs in 46 patients. Co-infused patients showed a faster and more robust hematopoietic recovery (Lazarus et al., 2005). However, contradictory results were obtained in patients transplanted with double UCB transplantation. In this study, the co-infusion of MSCs did not have any impact on the level of UCB HSPC engraftment or on aGvHD prevention, while MSCs resulted to be effective in treating aGvHD (Gonzalo-Daganzo et al., 2009), suggesting that further studies are necessary to define the proper dose and timing of MSC infusion to exploit their supportive function in the context of HSCT. Also, in the context of severe aplastic anemia, patients had an improved outcome after co-infusion of BM-MSCs in haploidentical HSCT (Liu et al., 2018). Recently, a phase I trial proposed intra-bone marrow transplantation of MSCs, in the CB transplantation setting, as a novel strategy to prevent GVHD and to increase donor cell engraftment (Goto et al., 2020). The safety and the prevention of GVHD demonstrated by this approach might be applied also in BM transplantation, especially in the case of HLA mismatches.

Additionally, the clinical effects of MSC co-transplantation have been also assessed in pediatric patients undergoing HSCT. Fourteen children enrolled in a phase I/II clinical trial showed an increased lymphocyte recovery and prevention of graft rejection (Ball et al., 2007) when co-infused with MSCs. In other studies, transplantation of *ex vivo* MSCs in children with acute leukemia showed promising results. Co-transplantation with third-party UCB MSCs resulted in early recovery of platelets and neutrophils without any engraftment failure among patients (Lee et al., 2013), and engraftment enhancement (Macmillan et al., 2009). Contrarily, another study involving 13 children with hematological diseases did not show any differences in terms of hematological recovery after the co-infusion of MSCs with UCB HSPCs, although prevention of aGvHD symptoms was observed, underlying the immunomodulatory and immunosuppressive effects of MSCs, which may be crucial to reduce transplanted-related mortality (Bernardo et al., 2011).

Importantly, the clinical benefits of MSCs may rely on their capacity to home toward the site of injury, that in the case of HSCT is the BM niche. It has been demonstrated that several factors may affect MSCs’ homing, including *ex vivo* culture, donor age, and route of administration (Pezzi et al., 2017). For instance, it has been shown that CXCR4, a chemokine receptor involved in MSCs migration, is downregulated upon *ex vivo* culture (Potapova et al., 2008). Moreover, several evidences suggest that the majority of MSCs infused intravenously remain trapped in the lung capillary network, and are subsequently distributed to other organs (Gao et al., 2001). On the contrary, other works demonstrated that MSCs home toward the injured tissues (Rustad and Gurtner, 2012), although the exact mechanisms are still largely understood. *In vivo* experiments have demonstrated that the MSCs reach the target damaged tissue thanks to the upregulation of inflammation-associated factors (Natsu et al., 2004; Spaeth et al., 2008). Nevertheless, it is still debated whether MSCs are capable of engrafting in the target tissue. In light of this, several strategies have been developed to favor MSC retention, including culture conditions, administration route, and genetic modifications of MSCs (De Becker and Riet, 2016).

Homing and engraftment of MSCs in the BM niche is an important aspect in the context of HSCT. The homeostasis of the BM niche is profoundly affected by the pre-transplant chemo- and/or radiotherapy (Arai et al., 2015). These results might perturb the cross-talk between HSPCs and the BM microenvironment that may lead to short- and long-term complications (Batsivari et al., 2020). MSC infusion might attenuate the damages induced by the conditioning, restoring the BM microenvironment, and as a consequence, facilitating the engraftment through the release of paracrine factors (Crippa and Bernardo, 2018).

In recent times, to overcome conditioning-related complications, novel and safer conditioning strategies have been developed. Antibody-based conditioning agents may represent an attractive alternative to the standard chemotherapy/radiotherapy as they specifically target HSPCs, sparing non-hematopoietic cells and reducing off-target toxicity (Palchaudhuri et al., 2016; Czechowicz et al., 2019). These monoclonal antibodies, coupled with toxins, have the potentiality to open to a greater application of HSCT and gene therapy approaches (Aiuti and Naldini, 2016).

In summary, the MSCs’ unique abilities to modulate the BM microenvironment, the immune system, and their hematopoietic supportive function render MSCs an attractive tool in the field of HSCT, becoming a promising and safe therapeutic candidate to treat immune-mediated disorders. Although MSC efficacy is variable among different reports, further studies and increased knowledge are required to better elucidate their mechanisms of action and to fully optimize MSC treatment. MSC dose, timing, and routes of administration are all aspects that require extensive investigation, in order to optimize their efficacy. In addition to pre-clinical and clinical studies aimed at confirming these promising results in a larger cohort of patients, it must be taken into consideration that MSC clinical application requires *in vitro* manufacturing. For this reason, culture condition protocols and expansion should be established with precise guidelines with the aim of maintaining MSC functional properties (Samsonraj et al., 2017).

## Discussion: Challenges and Perspectives for Mesenchymal Stromal Cells in the Context of Hematopoietic Stem Cell Transplantation

Mesenchymal stromal cells are key elements in the BM niche where they regulate HSPC homeostasis by direct contact and secreting several paracrine factors. In this review, we have discussed the mechanisms through which MSCs display a supportive effect on the BM niche and regulate HSPC homeostasis. Challenges and future perspectives on how to further improve MSC support to HSPCs in the context of HSCT are also reported.

Although several studies have clarified the identity of the MSC subset/s directly supporting HSPCs within the BM niche (Tormin et al., 2011; Crippa et al., 2019), the majority of available data describing the hematopoietic supportive activity of MSCs in the context of HSCT have been obtained using *ex vivo* expanded MSCs due to their low frequency in the BM (Li et al., 2016).

Unfortunately, prolonged passaging in culture induces the activation of a senescent program, which alters the gene expression profiling of *ex vivo* expanded MSCs (Banfi et al., 2002; Baxter et al., 2004). A total of 1,578 transcripts were differentially modulated in passage 10 compared with passage 2 MSCs. Upregulated genes belong to pathways involved in membrane integrity, receptor activity, vacuole, and lysosome, associated with the enlargement of the membrane compartment and vacuole formation in senescent cells. Downregulated genes are related to cell cycle, DNA replication, and repair mechanisms, following the reduced proliferation capacity observed in senescent MSCs (Wagner et al., 2008). A significant reduction in CD146^+^ MSC fraction has been also observed, highlighting the loss of primitive progenitors after prolonged expansion (Jin et al., 2016; Gnani et al., 2019). The activation of a senescent program functionally impaired the differentiation potential of BM-MSCs, with some studies suggesting an unbalanced adipogenic differentiation at the expense of osteoblastogenesis (Stolzing et al., 2008; Yang et al., 2018). In addition, aging MSCs acquire a senescence-associated secretory phenotype, which alters the composition of their secretome. Higher production of pro-inflammatory cytokines has been observed in MSCs expanded *in vitro* for several passages (Turinetto et al., 2016). Similarly, IL6, IL8, and MCP1 were shown to be increased in the conditioned medium of aged MSCs (Gnani et al., 2019), potentially affecting the beneficial effects of MSC paracrine activity. More specifically, the release of inflammatory cytokines associated with prolonged *in vitro* culture could impair the anti-inflammatory and immunoregulatory activity of MSCs (Sepulveda et al., 2014). Moreover, the secretion of senescence-associated secretory phenotype (SASP) proteins not only reinforces the establishment of a senescent program in a cell-autonomous manner but also affects the physiology of neighboring cells. For example, it has been shown that HSPCs’ clonogenic capacity decreases when co-cultured with aged MSCs (Gnani et al., 2019).

Extensive *in vitro* culture induces the activation of a DNA damage response altering the functional properties of MSCs. This is mainly due to the accumulation of ROS associated with the oxidative metabolism of cultured cells and to the downregulation of genes involved in DNA repairing (Bao et al., 2020). Indeed, a decrease in the number of repairing γH2AX/53BP1 DSB repair foci was observed in long-term culture of MSCs, in addition to a slower DNA repair kinetics, and an increased number of residual DNA double-strand breaks 7 h post irradiation. This leads to chromosomal instability, increasing the risk of genetic transformation in culture (Hladik et al., 2019). Exposure to etoposide (VP16) was utilized as an assay to determine when *ex vivo* expanded MSCs display impaired DNA repair abilities, indicating the need to analyze the DNA repairing machinery and possible genetic aberration in MSCs intended for transplantation (Hare et al., 2016).

These data highlighted the need to develop specific and validated culture systems to maintain MSC biological and functional characteristics when expanded *in vitro* for clinical purposes. With this aim, the use of three-dimensional structure has been shown to improve the preservation of the therapeutic potential of MSCs compared with traditional 2D culture expansion. 3D cultures demonstrated enhanced potential for adipogenic and osteogenic differentiation (Banfi et al., 2000, 2002; Bae et al., 2017; Tietze et al., 2019). Of note, the genomic profiling of MSCs cultured in spheres showed a gene expression pattern comparable to primary freshly isolated MSCs, demonstrating that non-adherent cell culture conditions better preserve the biological and functional characteristics of MSCs (Ghazanfari et al., 2017). 3D co-culture with MSCs significantly improved the self-renewal and *in vitro* proliferation of HSPCs (Huang et al., 2016; Costa et al., 2019; Raic et al., 2019).

**FIGURE 3:**
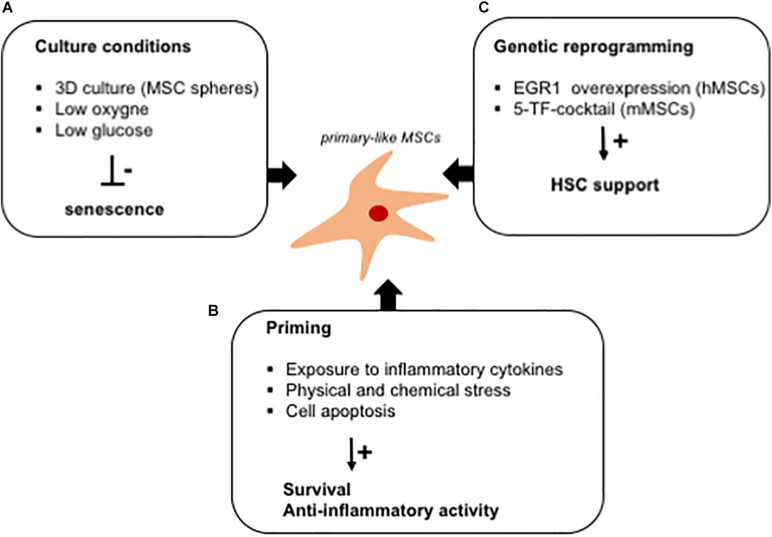
*In vitro* strategies to fully exploit the hematopoietic supportive capacity of *ex vivo* expanded MSCs. Schematic representation of culture strategies aimed at improving the hematopoietic supportive capacity of MSCs, which is impaired upon *ex vivo* expansion. **(A)** Several strategies (mesensphere, low oxygen, and low glucose cultures) have been developed to avoid the activation of a senescence program altering the secretory phenotype of *ex vivo* expanded MSCs. **(B)** MSC priming with inflammatory cytokines has been demonstrated to increase the release of anti-inflammatory cytokines by MSCs. Similarly, exposure to culture stress increases the survival rate of MSCs when transplanted *in vitro*. The immunomodulatory activity of MSCs has been exploited for the treatment of GVHD by inducing cell apoptosis in *ex vivo* expanded cells. **(C)** *Ex vivo* reprogramming by the overexpression of specific transcription factors has been studied as a strategy to render *ex vivo* expanded MSCs similar to primary cells, with a potentiated hematopoietic supportive ability.

Several strategies have also been tested to reduce the culture-induced replicative stress in *ex vivo* expanded MSCs, including the control of glucose supply, the addition of proper cytokines or growth factors, and the regulation of oxygen levels (Figure 3). Within the BM niche, oxygen supply is much lower than in culture, leading to increased production of ROS and oxidative stress in *ex vivo* expanded cells. This has been shown to induce the activation of a DNA damage response, causing the establishment of an early senescence program impairing MSCs’ functionality. As an example, excessive ROS activate the peroxisome proliferator-activated receptor-gamma (PPARγ), favoring adipogenesis in *ex vivo* expanded MSCs (Atashi et al., 2015). In the presence of high ROS levels, the hematopoietic supportive capacity of MSCs is impaired leading, for example, to a reduced capacity to support the *ex vivo* expansion of HSPCs, which, in turn, fail to form a proper and functional BM niche when used in humanized ossicle models (Crippa et al., 2019a). The expansion of MSCs in low-oxygen conditions increases their proliferation capacity, efficiently improves the differentiation potential into mesodermal cell types, and promotes their survival when transplanted (Estrada et al., 2012). Hypoxic culture conditions improve growth kinetics, genetic stability, and expression of chemokine receptors during *in vitro* expansion and could eventually enhance the therapeutic efficacy of MSC-based therapies (Haque et al., 2013; Hu and Li, 2018). In accordance, the expansion of MSCs in a high-glucose medium impairs their proliferation capacity and induces the activation of a premature senescence program due to a metabolic switch toward an oxidative metabolism (Li et al., 2007). In light of this, the antioxidant activity of glutathione has been shown to improve the efficacy of infused MSCs in the control of aGvHD (Antebi et al., 2018; Lim et al., 2020).

Furthermore, several studies have identified specific priming strategies to enhance the therapeutic efficacy of *ex vivo* expanded cells (Noronha et al., 2019). In particular, MSCs have been exposed to inflammatory cytokines to induce the activation of an anti-inflammatory program, which improves their capacity to counteract the progression of inflammatory and autoimmune diseases (Domenis et al., 2018; Lin et al., 2019). In the context of aGvHD, the apoptosis of infused MSCs, induced by recipient cytotoxic cells, triggers a cascade signaling, which governs immune cells to counteract the disease progression. The infusion of MSCs, induced *in vitro* to enter apoptosis, has been proposed as a strategy to enhance the immune-regulatory activity of MSCs, in a mouse model of aGvHD (Galleu et al., 2017). Similarly, MSC-derived apoptotic bodies have been shown to control macrophage polarization for tissue repair (Liu et al., 2020). Also, MSC preconditioning with physical or chemical stress enhances their survival when transplanted, by inducing the expression of pro-survival and anti-apoptotic factors (Pasha et al., 2008; Raziyeva et al., 2020).

The need to develop culture strategies to restore the functional properties of MSCs has recently arisen due to an impairment in MSC function caused by plastic adherence and exposure to culture media. An important aspect to consider for the clinical use of MSCs in the context of HSCT is that *ex vivo* expansion reduces the hematopoietic supportive activity of MSCs and impairs their capability to home back into the BM niche when infused. *In vitro*, MSCs become a heterogenous population, with the establishment of different MSC subpopulation characterized by specific functional properties, including self-renewal capacity and differentiation potential (Tormin et al., 2009; Whitfield et al., 2013). RNA sequencing analysis of *ex vivo* expanded and primary MSCs demonstrated that the global gene expression profiling of primary cells differs from that of cultured cells (Qian et al., 2012; Ghazanfari et al., 2017). Specifically, the expression of genes involved in immune response, mRNA processing, and antigen presentation is significantly reduced upon *ex vivo* culture compared with that of cells prospectively isolated from the human BM as Lin^–^/CD45^–^/CD31^–^/CD71^–^/CD235^–^/CD271^+^ cells. Several transcription factors were also found differentially expressed in *ex vivo* MSCs compared with primary cells. The majority of these regulate stem cell functions, including FOS, FOSB, HMGB3, EGR1, PPARG, ATF4, NFE2L1, and SOX4 (Ghazanfari et al., 2017). Among these, EGR1 has been studied in human MSCs due to its function in regulating MSCs’ paracrine activity inducing the release of hematopoietic supportive factors (Tamama and Barbeau, 2012; Li et al., 2020). In particular, it has been shown that human *ex vivo* expanded MSCs, engineered to overexpress EGR1, sustain HSPC expansion and maintenance in 2D co-culture more efficiently than control cells (Li et al., 2020; Figure 3). These results correlate with more robust engraftment of long-term repopulating HSPCs into a pre-clinical model of transplantation. This aspect is particularly relevant when proposing engineered MSCs as a tool to optimize the efficacy of HSCT.

Another important aspect to consider for a proper clinical use of MSCs is the method used to preserve and pool cells, while expanding to reach the proper number for clinical application. Slow freezing is the most common cryopreservation method to freeze a large number of MSCs at a low concentration, with a freezing rate of 1°C/min and in the presence of DMSO as cryoprotective agent. However, the risk of freeze injury altering the functional properties of MSCs is very high. For this reason, several systems have been recently developed to better preserve MSCs, including a programmed freezer (Cell Alive System), which vibrates the water molecules and cells using alternating magnetic field and electric field during the process of freezing to prevent intra- and extra-cellular ice formation and novel cryoprotective agents, less toxic, such as non-toxic polymers (e.g., polyvinylpyrrolidone) and disaccharide (Janz Fde et al., 2012; Renzi et al., 2012; Rogulska et al., 2019). Several functional differences were found in thawed MSCs compared with fresh MSCs at earlier readout post-thawing due to the activation of a cellular heat shock response, which is recovered after extensive *ex vivo* culture (Francois et al., 2012a; Moll et al., 2014). Genomic alterations were also observed in MSCs post-thawing (Hoogduijn et al., 2016). In light of these, several studies are focused on defining better thawing conditions to increase cell viability (apoptosis inhibition and preservation of membrane integrity) and to accelerate the restoration of MSC functional characteristics (heat shock protein activation before thawing) (Baust et al., 2000; Malpique et al., 2009; Shaik et al., 2017).

The use of MSC-derived secretome, as a lyophilized medical product, could overcome any concerns regarding the infusion of *ex vivo* expanded cells, thawed or genetically manipulated. The therapeutic use of MSCs’ secretome offers several advantages compared with MSC infusion. Secretome is generally considered safer than cells: it lacks the potential for endogenous tumor formation as it cannot self-replicate, it has low immunogenicity, and leads to low risk of emboli formation when intravenously injected (Teixeira et al., 2013; Bari et al., 2018). The use of MSC-secretome in therapy also includes several technological advantages: it can be manipulated and stored as a ready-to-use product easier and cheaper than cells (Harrell et al., 2019). Of note, the efficacy of MSC-secretome administration has been demonstrated in the context of different pathologies, including pulmonary disease (Bari et al., 2019, 2020), neurodegenerative disorders (Santamaria et al., 2020), cutaneous wound healing (Park et al., 2018; Ahangar et al., 2020), cardiovascular disease (Ranganath et al., 2012), and liver conditions (Driscoll and Patel, 2019).

Similar to human MSCs, murine cells show a reduced hematopoietic supportive capacity *in vitro*. A cocktail of five transcription factors has been identified to reprogram murine MSCs into primary-like cells, characterized by an enhanced hematopoietic supportive activity (Figure 3). The synthesis of HSPC supportive factors is significantly higher in reprogrammed cells while maintaining their differentiation potential. It has been demonstrated that HSPCs co-cultured in the presence of reprogrammed MSCs show an improved long-term repopulating capacity and enhanced hematological reconstitution capability when transplanted, compared with HSPCs expanded on control MSCs (Nakahara et al., 2019). Analysis of phosphorylated gH2AX, a marker of DNA damage response activation, showed that reprogrammed MSCs efficiently protect HSPCs from accumulating DNA damage, associated with replicative stress. Similar results were obtained for human HSPCs expanded on reprogrammed murine MSC feeder. Collectively, these results highlight the possibility to exploit the hematopoietic supportive capacity of human MSCs by genetic reprogramming and, eventually, to propose the use of reprogrammed MSC-derived secretome to ameliorate the outcome of HSCT (Nakahara et al., 2019). This direction appears highly attractive for those conditions requiring significant cell expansion due to a low number of HSPCs available for transplantation or when *ex vivo* genetic manipulation is necessary, such as for gene therapy and gene-editing protocols. These methods lead to HSPCs’ distress by the activation of a DNA stress response due to the *ex vivo* gene manipulation procedures, which result in a reduction of the long-term repopulating capacity of gene-corrected HSPCs (Schiroli et al., 2019).

The capacity to home and engraft in the BM niche is one of the MSCs’ functional characteristics impaired by the *ex vivo* expansion. Several studies have shown that only a small percentage of MSCs reach the BM (Leibacher and Henschler, 2016) when systemically administered in co-infusion protocols of HSCT. This limits their hematopoietic support and precludes the possibility to repair the stromal niche from conditioning-associated damage or disease-associated alterations (Amarachintha et al., 2015; Starc et al., 2017; Crippa et al., 2019, 2019a; Severe et al., 2019). Although the clinical benefits of MSCs are based on their capacity to release supportive and anti-inflammatory factors, their activity promoting HSPC engraftment would be more efficient in the case of local release. However, the BM vasculature damage induced by chemo-radiotherapy may reduce the homing efficiency of MSCs (Chen et al., 2019), in addition to the fact that *ex vivo* expansion alter the MSC motile properties (Dorland et al., 2019). Overexpression of CXCXR4 and VLA-4 has been explored as a strategy to improve the homing and survival of infused MSCs in pre-clinical models of HSCT (Kumar and Ponnazhagan, 2007; Chen et al., 2013; Ullah et al., 2019). Different from *ex vivo* expanded MSCs, freshly isolated cells are able to engraft in the BM niche and repair the niche damage, induced by the conditioning regimen. The intra-bone transplantation of primary murine MSCs restored the stromal component of the niche and significantly improved the outcome of HSCT compared with the infusion of *ex vivo* expanded cells (Abbuehl et al., 2017). The functionality of the supportive stromal niche is a key aspect for the proper engraftment and survival of long-term repopulating HSPCs. Conditioning regimens have several off-target effects impairing the supportive activity of the BM stroma and inducing the release of several inflammatory cytokines that could alter the fate of transplanted HSPCs. For example, it has been shown that IL1 induces premature myeloid differentiation of HSPCs (Pietras et al., 2016). Alterations in the BM niche have been also reported in specific pathological contexts, for which HSCT is the only curative option. In patients affected by β-thalassemia, MSCs show a reduced hematopoietic supportive capacity, associated with increased levels of ROS derived from the continuous exposure to iron overload (Crippa et al., 2019a). Furthermore, a reduced anti-inflammatory capacity has been observed in MSCs isolated from patients affected by immunological disorders (Starc et al., 2017). In the case of hematological malignancies, the functionality of the BM niche is also altered (Mendez-Ferrer et al., 2020). In all these conditions, restoring a proper supportive function is fundamental to ameliorate the outcome of HSCT.

## Conclusion

In conclusion, definition and validation of *ex vivo* culture methods capable to best preserve MSCs’ biological and functional properties, genetic manipulation of *ex vivo* expanded MSCs possibly enabling them with primary cell-like functions, and use of MSC-derived secretome represent fascinating approaches to further exploit the hematopietic supportive and immunoregulatory activities of MSCs in support of HSCT.

## Author Contributions

SC wrote and revised the manuscript. MB, LS, and GD wrote the manuscript. MB supervised and revised the work. All authors contributed to the article and approved the submitted version.

## Conflict of Interest

The authors declare that the research was conducted in the absence of any commercial or financial relationships that could be construed as a potential conflict of interest.
